# Friend or Foe? Flipped Classroom for Undergraduate Electrocardiogram Learning: a Randomized Controlled Study

**DOI:** 10.1186/s12909-017-0881-8

**Published:** 2017-03-07

**Authors:** Zeng Rui, Xiang Lian-rui, Yue Rong-zheng, Zeng Jing, Wan Xue-hong, Zuo Chuan

**Affiliations:** 1 Department of Cardiovascular Diseases, West China Hospital, School of Clinic Medicine, Sichuan University, Chengdu, China; 2 Department of Public affairs development, West China Hospital, School of Clinic Medicine, Sichuan University, Chengdu, China; 3 Department of Nephrology, West China Hospital, School of Clinic Medicine, Sichuan University, Chengdu, China; 4 Department of Internal Medicine, West China Hospital, School of Clinic Medicine, Sichuan University, Chengdu, China; 5 Department of Rheumatology and Immunology, West China Hospital, School of Clinic Medicine, Sichuan University, Chengdu, China

**Keywords:** Flipped classroom, Lecture-based learning, Electrocardiogram learning, Medical education

## Abstract

**Background:**

Interpreting an electrocardiogram (ECG) is not only one of the most important parts of clinical diagnostics but also one of the most difficult topics to teach and learn. In order to enable medical students to master ECG interpretation skills in a limited teaching period, the flipped teaching method has been recommended by previous research to improve teaching effect on undergraduate ECG learning.

**Methods:**

A randomized controlled trial for ECG learning was conducted, involving 181 junior-year medical undergraduates using a flipped classroom as an experimental intervention, compared with Lecture-Based Learning (LBL) as a control group. All participants took an examination one week after the intervention by analysing 20 ECGs from actual clinical cases and submitting their ECG reports. A self-administered questionnaire was also used to evaluate the students’ attitudes, total learning time, and conditions under each teaching method.

**Results:**

The students in the experimental group scored significantly higher than the control group (8.72 ± 1.01 vs 8.03 ± 1.01, t = 4.549, *P* = 0.000) on ECG interpretation. The vast majority of the students in the flipped classroom group held positive attitudes toward the flipped classroom method and also supported LBL. There was no significant difference (4.07 ± 0.96 vs 4.16 ± 0.89, Z = − 0.948, *P* = 0.343) between the groups. Prior to class, the students in the flipped class group devoted significantly more time than those in the control group (42.33 ± 22.19 vs 30.55 ± 10.15, t = 4.586, *P* = 0.000), whereas after class, the time spent by the two groups were not significantly different (56.50 ± 46.80 vs 54.62 ± 31.77, t = 0.317, *P* = 0.752).

**Conclusion:**

Flipped classroom teaching can improve medical students’ interest in learning and their self-learning abilities. It is an effective teaching model that needs to be further studied and promoted.

**Electronic supplementary material:**

The online version of this article (doi:10.1186/s12909-017-0881-8) contains supplementary material, which is available to authorized users.

## Background

Medical Diagnostics is one of the core courses in the higher medical education curriculum in China. It is an important bridging course and provides a transition from basic to clinical medicine. This course covers medical history, physical examinations, laboratory tests and examinations, and the rationale behind a clinical diagnosis. This course aims to teach learners to apply basic medical theory, basic medical knowledge, and basic medical skills for the analysis, judgment, diagnosis, and classification of diseases. Although the traditional LBL method used for teaching Medical Diagnostics is effective for learning a significant part of the content, this method has also been criticized by many researchers as being ineffective to help students acquire the necessary knowledge and skills. When used as the only teaching method, it has negative effects because the students are passive during the lecture and have almost no time to take the initiative to understand, think, and develop problem-solving skills [1, 2]. Moreover, the students have almost no opportunity to receive individualized instruction or experience independent learning, because communicating the theoretical knowledge and basic principle contents take up a significant amount of the classroom teaching time. Furthermore, the teachers are also unable to meet the specific demands of each student during such sessions [3, 4]. Thus, it is important to explore methods that have the potential to maximize the use of classroom time and transform the classroom into a platform for teacher-student interactions and student thinking.

The flipped classroom has emerged in the context of the widespread use of information technology and the Internet, and it complements the traditional teaching models [5, 6]. As early as 1996, a study by Lage et al. from the School of Business at the University of Miami proposed an “inverted classroom” and a change in the traditional order of pre-class and in-class studies [5]. Subsequently, Wesley Baker proposed a “classroom flipping” model [6]. Baker described the nature of flipped learning for the first time and emphasized that the teacher was no longer the authority on the podium (“Sage on the Stage”) in a flipped classroom but had instead become a mentor for the students (Guide on the Side). In the flipped teaching mode, students view the video-on-demand according to their knowledge levels. They can selectively “listen” to the lecture but can also replay key and difficult content. In the classroom, time is sufficiently and appropriately used for face-to-face discussions between the teachers and students. These discussions are focused on improving understanding of the core and difficult parts of the course, at times about controversial topics and for promoting the migration and application of knowledge [7]. The flipped classroom provides the option of transferring knowledge outside the classroom as well, thereby allowing students to freely choose the most suitable method to acquire knowledge. This model consigns the process of integrating knowledge to class time, in order to enhance interactions and deepen collaborations among students and between students and the teacher. This system converts the passive acceptance in traditional classroom learning into self-exploration by respecting the individual characteristics of cognitive learning [7].

Recently, the flipped classroom has been adopted by an increasing number of medical schools [7–14]. EA van Vliet et al. found that flipped-class pedagogy enhanced student metacognition and collaborative-learning strategies [7]. Gorres-Martens BK et al. also revealed that positive outcomes increased over time with the implementation of a flipped teaching model [9], while Bossaer JB et al. found that the students who experienced the flipped classroom approach performed poorer on examination questions compared with the lecture cohort in a pharmacotherapy oncology module [8]. This poses the research problem of gauging the advantages and disadvantages of using the flipped classroom method for medical education—determining whether it is a friend or foe. Further research is needed to determine the efficiency of the flipped classroom teaching method for medical teaching.

To further develop this context, we introduced flipped classroom teaching into the teaching of Medical Diagnostics. The topic of ECG in Medical Diagnostics was selected as a research subject because it is a standalone unit containing relatively independent content, which would ensure that few distractions affect the study results; however, it is also an abstract principle of knowledge that is difficult to understand, and requires a high level of application. We sought to observe whether flipped classroom teaching improved the effects of teaching in the limited time period, and investigated the students’ and teachers’ attitudes towards the flipped classroom. We were also interested in exploring the feasibility and value of the flipped classroom for the teaching of Medical Diagnosis, and aimed to provide a theoretical basis for a wider range of its applications.

## Methods

### Study design

A randomized controlled trial (RCT) study was performed with students as the research participants. A total of 181 junior-year students majoring in clinical medicine participated in this study during the academic year 2015–16. The students were further grouped into two groups using a computer-based random digital method. Ninety students were in the flipped classroom group, whereas the control group included ninety-one students who were taught using the LBL method.

### Teaching method

The two groups of students received the same textbook [15], the same syllabus, and the same practical guidance. They also had the same instructors, teaching schedule, and examination format. In the ECG chapter, the respective groups were taught using flipped classroom teaching or LBL. The ECG chapter consisted of three classroom units for which classes were held over three consecutive weeks (Table 1). The following specific teaching methods were used:

**Table 1:** Contents of ECG intepretation

Analysis content	Normal	Abnormal
Heart rate	60–100per/min	<60per/min >100per/min
Heart rhythm	Regular	Irregular
P wave	Sinus P-wave	Non-sinus P-wave
PR interval period	0.12–0.20 s	<0.12 s >0.20 s
QRS wave	Normal QRS wave	Abnormal voltage
		Abnormal electric axis
		QRS duration augmentation
		Pathological Q wave
ST segment	Normal ST segment	Elevation and depression of ST segment
T-wave	Normal T wave	Tip, flat or inverted T wave
Other issues:		U-wave
		Abnormal electrolyte related ECG
		Drugs related ECG

#### Flipped classroom method (see Fig. 1)

**Fig. 1:**
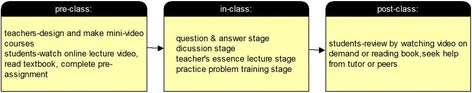
Flowchart of the flipped class structure and settings

1. Pre-class preparation phase
  A. Teacher preparation: One year prior to the ECG test, the teachers were trained for microteaching sessions. The teachers then performed decomposition and refined their knowledge, compiled the script, produced micro-video lessons verified by a team of experts, and uploaded the videos online. Each video was accompanied with questions and exercises to guide self-learning and test learning outcomes.
  B. Student preparation: Ten days prior to the classes, the teachers clarified the purpose of the teaching and curriculum to the students, informed the students about the procedures and requirements of flipped classroom learning and obtained written informed consent from the students. One week before each teaching unit was administered, teaching assistants announced the links on the micro-lesson learning platform and discussion site and issued reading materials, including textbook sections, PowerPoint courseware, supplementary teaching materials for the ECG, and ECG exercise information. The teaching assistants divided the students into 8 groups by computer-based random digital method, with 11–12 students per group. The groups chose their leaders who determined the time for micro-lesson learning and coordinated their respective teams to address the questions and exercises. During the learning period, the teams were free to discuss the online course content with their own teams. One day before the class for each teaching unit, the teaching assistants collected the assignments and exercises and submitted them to the instructors.
  C. Equipment: The teaching assistants booked classrooms with tablets and a network interface in advance and tested the voting machines and other equipment.
2. Classroom phase
  The 135 min for each teaching unit was divided into four stages. In the first stage, teachers attempted to clarify any doubts that the students had. Based on the assignments and discussions on the website, the teachers spoke about on the difficulties and problems exposed during student learning. This stage lasted approximately 20 min. The second stage was an interactive discussion between the teachers and students. The students freely asked questions that could be answered either by other students or by the teacher. This stage lasted approximately 40 min. In the third stage, the teacher gave a succinct lecture. The teacher emphasized the major and difficult aspects of this unit. Depending on whether students raised any concerns and based on their feedback, the teacher briefly asked questions after key points or offered a teacher-student interaction session, such as time for questions and answers. This stage lasted approximately 30 min. In the fourth stage, the students performed exercises. The teacher presented an ECG, and the groups competed to analyse the graphs. The group that won the right to answer sent a representative to explain the ECG; the other teams could provide supplementary answers, ask questions, or participate in discussions. The teacher provided simple reviews. Finally, the groups anonymously voted for the group that contributed the most to a correct interpretation (including the group that provided the key analysis and the group that contributed to the interpretation and supplementary discussion). This stage included discussions of 8–10 ECGs and lasted for approximately 45 min.
3. After-school review
  After the lecture, the students replayed the micro-video lessons to review difficult points according to their individual ability to understand the content. They were also able to use the course platform for group study and could ask the teacher, or teaching assistant, questions.

#### LBL method

The teaching assistant of the control group distributed reading materials prior to class, including the corresponding textbook chapters, electrocardiogram supplementary teaching materials, and ECG exercises. During class, the LBL method was used as followed. Each teaching unit contained a 125-min lecture followed by approximately 10 min of answering questions. After class, the teaching assistant provided the PPTs used for holding the lecture. Prior to and after class, the students were free to learn based on their preference.

## Evaluation methods

One week after the topic was presented using respective teaching methods, both groups received a test to assess their understanding of the content. The assessment included questions concerning ECG interpretation that were prepared in advance by the teachers. The assessment primarily tested the ability of the students to analyse and diagnose typical lesions on an ECG. The test contained 10 ECG questions. Some questions contained a short summary of medical records and required the students to make a comprehensive and accurate ECG diagnosis. The examinations were held in a Computer-Assisted Instruction (CAI) classroom on a computer. The different groups of students received the same questions but in a different order. Two minutes were given for students to answer each question, and the total time was twenty minutes. The two test papers were previously assessed to ensure consistency in difficulty levels. The test scores of the two groups were compared and statistically analysed.

A self-administered questionnaire was used to evaluate the students’ attitudes and learning experiences under each teaching method. The opinions about the flipped classroom were only collected from the test group. The questionnaire contained 14 entries and was graded using a five-point Likert scale with 1 indicating “very dissatisfied” and 5 indicating “very satisfied” [16]. The total time of learning and other related information between the two groups were collected using fill-in-the-blank questions in the questionnaire.

We also conducted semi-structured interviews of teachers to understand their experiences of both the flipped classroom and LBL method. Teachers who participated in the interviews talked about the time they spent and other learnings. In addition, they were asked to provide a subjective evaluation of the teaching atmosphere and the perceived effects of the respective teaching method they used. Twenty teachers were interviewed, and they were recorded using videos. Examples of questions include the following: How much time did you spend? What were the other types of learning trainings? What is your opinion of the flipped classroom and why? Based on the information conveyed in the video recordings of the interviews, we calculated the total average time spent and categorized the interview responses based on similarities. You could find the detailed information for interview questionnaire on teacher’s attitudes towards the two pedagogics in the Additional file 1.

### Statistical analysis

The results were analysed using the SPSS 17.0 statistical software package. The data are presented as the mean ± sd or in percentages with α = 0.05 as the level of significance. The data were first subjected to the Kolmogorov-Smirnov normal distribution test after which the variance was tested using Levene’s Test for Equality of Variances. Data that passed the test were subjected to a *t*-test while data with non-normal distributions were tested using the rank-sum test. The test scores of the two groups after the teaching intervention and the time invested by the two groups of students were compared with an independent sample *t* test. The scores of the questionnaire results were compared using the Wilcoxon signed-rank test.

## Results

### Baseline student characteristics

A total of 181 students were enrolled in our study. All the participants had provided their consent. There were no significant differences between the two groups in terms of baseline variables, such as age, gender ratio or core course grade point averages in the past year (See Table 2).

**Table 2:** Baseline student characteristics

Groups	Age	Gender (Male/Female)	Anatomy	Pathophysiology	Pathology
flipped group (*n* = 90)	20.84 ± 0.67	41/49	77.86 ± 10.53	81.09 ± 10.63	78.47 ± 10.41
control group (*n* = 91)	20.90 ± 0.58	50/41	80.16 ± 9.37	79.43 ± 10.62	78.87 ± 11.13
t or *χ2*value	0.610	1.596	−1.559	1.051	−0.251
*P* value	0.543	0.207	0.121	0.294	0.802

### Comparison of ECG scores of the two groups

**Fig. 2:**
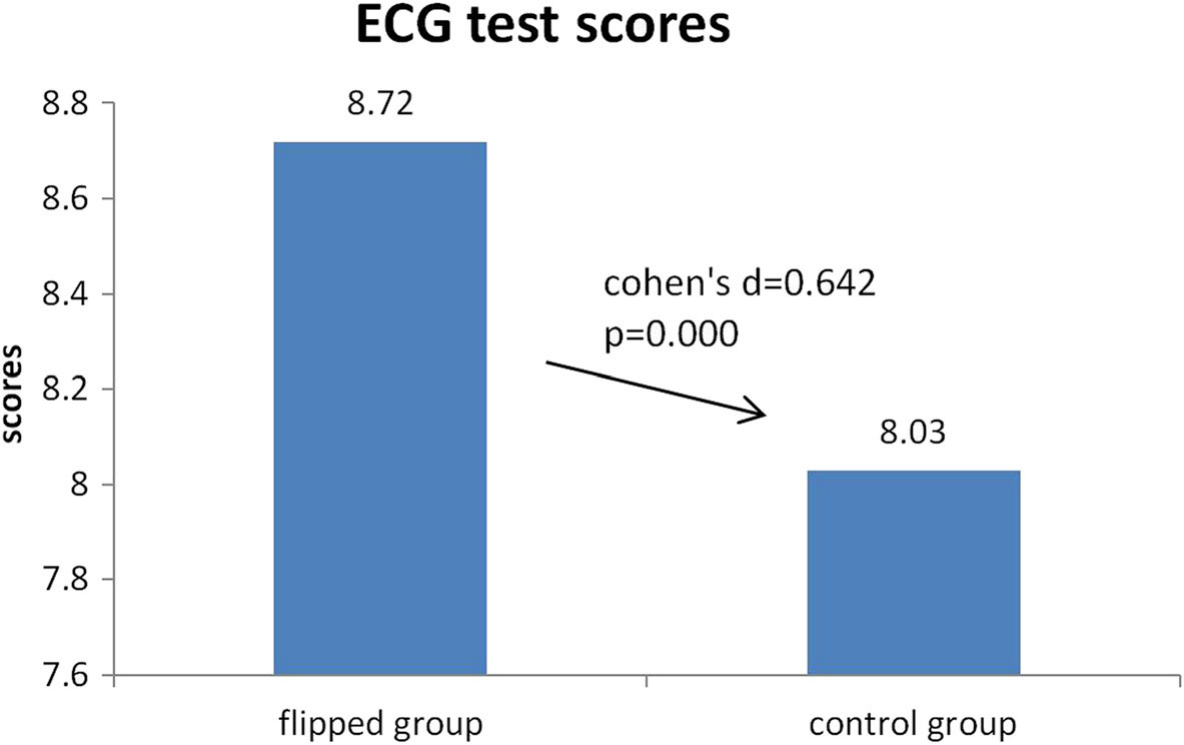
ECG test score differences between flipped class group and control group. The ECG test was administered one week after the classes. Students in the flipped class group scored significantly higher than the control group (8.72 ± 1.01 vs 8.03 ± 1.01, t = 4.549, *P* = 0.000)

The ECG interpretation test was administered a week after the topics were communicated via the respective method to each group. The students in the flipped classroom group scored significantly higher than the control group (8.72 ± 1.01 vs 8.03 ± 1.01, t = 4.549, *P* = 0.000). See Fig. 2 for details.

### Students’ attitudes towards the flipped classroom

A total of 181 questionnaires were returned with a return rate of 100%. The ninety questionnaires from the flipped classroom group revealed that the vast majority of the students held positive attitudes toward the flipped classroom and also supported the LBL method. There was no significant difference (4.07 ± 0.96 vs 4.16 ± 0.89, Z = − 0.948, *P* = 0.343). Most students chose “agree or strongly agree” on the following entries: “The flipped classroom stimulated interest in learning,” “The flipped classroom was helpful in my self-directed learning,” “I benefited from the teacher’s lecture on key points,” “I benefited from the question-and-answer sessions by teachers in the flipped classroom,” and “I benefited from class discussion with peers.” These items received, on average, higher than four points on the five-point Likert scale. Students awarded “I benefited from the teacher’s lecture on key points” and “I benefited from the question-and-answer sessions by teachers in the flipped classroom” significantly higher scores than “I benefited from class discussion with peers” (z = 2.587, *P* = 0.010 & z = 3.138, *P* = 0.002). The majority of students agreed that “The delivery of knowledge in a flipped classroom is fragmented and unsystematic” and that “The flipped classroom brought an increase in workload.” Refer to Table 3 for further details.

**Table 3:** Students’ feedback on the teaching model

Items	Score (Mean ± standard error)	Percentage of responding ≥4 (n,%)
1. Your attitude to the traditional ECG teaching mode	4.16 ± 0.89	68 (75.56%)
2. Your attitude to the micro-video lessons + flipped classroom	4.07 ± 0.96	61 (67.78%)
3. The flipped classroom stimulated interest in learning	4.01 ± 1.04	58 (64.44%)
4. The flipped classroom was helpful in my self-directed learning	4.1 ± 1.03	65 (72.22%)
5. I can watch or selectively watch the micro-video lessons according to my own situation any time	4.24 ± 0.92	72 (80.00%)
6. The flipped classroom was helpful in mastery of knowledge	4.09 ± 0.89	64 (71.11%)
7. I benefited from watching micro-video lessons before class	4.22 ± 0.93	71 (78.89%)
8. I benefited from the teacher's lecture on key points	4.43 ± 0.81	77 (85.56%)
9. I benefited from class discussion with peers	4.2 ± 0.99	68 (75.56%)
10. I benefited from the question and answer sessions by teachers in the flipped classroom	4.43 ± 0.78	76 (84.44%)
11. I benefited from watching micro-video lessons for some key pointsafter class	4.3 ± 0.89	71 (78.89%)
12. The flipped classroom brought an increase in workload	3.9 ± 1.03	52 (57.78%)
13. The delivery of knowledge in a flipped classroom is fragmented and unsystematic	3.88 ± 1.04	54 (60.00%)
14. The flipped classroom is an effective teaching model that is worthy of promotion.	4.17 ± 1.07	65 (72.22%)

### Comparison of the investment in studies between the two groups

**Fig. 3:**
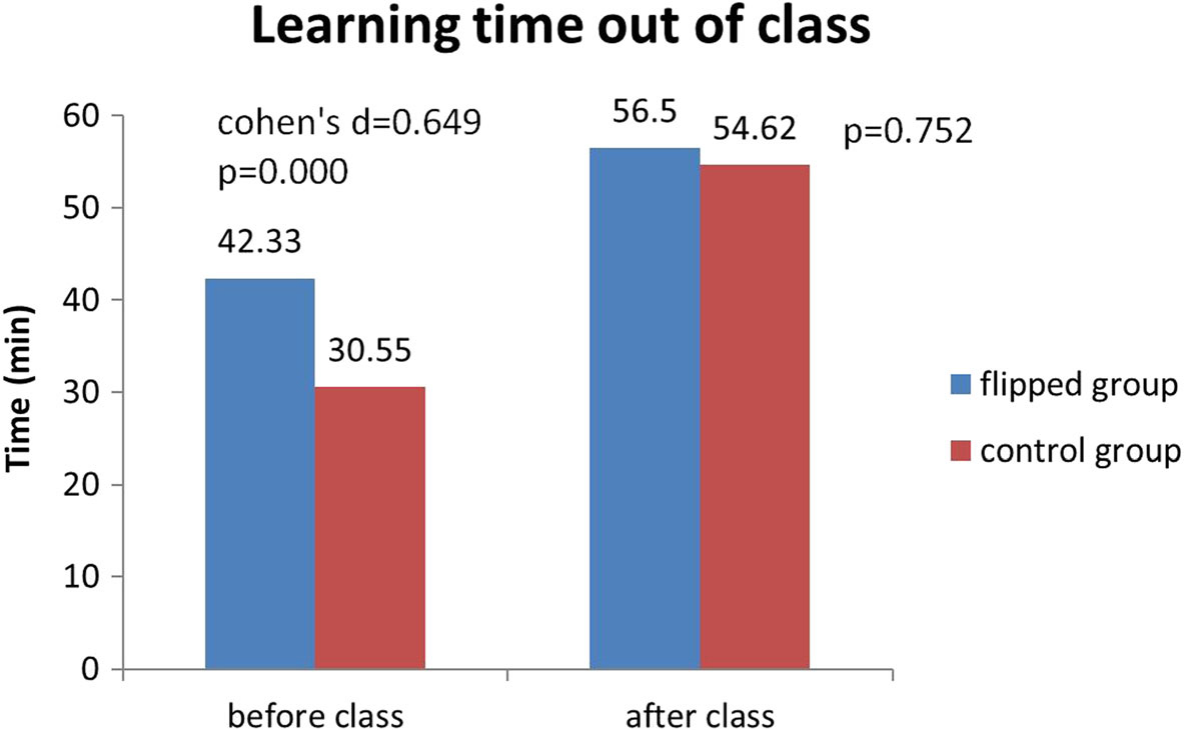
Differences in learning time out of class between flipped class group and control group. Prior to the class, the students in the flipped class group devoted significantly more time than those in the control group (42.33 ± 22.19 vs 30.55 ± 10.15, t = 4.586, *P* = 0.000), whereas after the class, the time spent by the two groups were not significantly different (56.50 ± 46.80 vs 54.62 ± 31.77, t = 0.317, *P* = 0.752)

Prior to the class, the students in the flipped class group devoted significantly more time to learning than those in the control group (42.33 ± 22.19 vs 30.55 ± 10.15, t = 4.586, *P* = 0.000). However, after class, the times spent by the two groups were not significantly different (56.50 ± 46.80 vs 54.62 ± 31.77, t = 0.317, *P* = 0.752), as shown in Fig. 3. The survey showed that the students in the flipped classroom group experienced more diverse learning. The group exposed to LBL usually only read the course material, PPT courseware and class notes for preview and review; however, the students exposed to flipped classroom teaching method used study methods such as finding information in library databases, discussions with peers, repeated studying using instructional videos and other network resources, and expressed the initiative to ask teachers and teaching assistants questions.

### Interview on teacher’s attitudes towards the flipped classroom and investment in the two pedagogics

The teachers who participated in the flipped classroom stated in their interviews that they invested a significant amount of time and effort in the micro-video production preparatory stage of the flipped classroom (120 h on average). Additionally, all teachers visited many other medical universities in China and Singapore in order to learn how to organize flipped classroom activities, or participated in several seminars about flipped classroom. For the classroom stage, teachers spent more than 30 h on average preparing before the class and 6 h on average after the class in flipped classroom teaching, whereas those who engaged in the LBL method spent only 10 h on average for the preparation and classroom stage. Although teachers’ investment, in terms of both time and energy, for the flipped classroom was considerably more than for the LBL method, teachers considered the teaching atmosphere, interest, and enthusiasm towards learning in the flipped classroom to have substantially improved, and considered it worth the additional effort.

## Discussion

The results of the students’ scores indicated that the flipped classroom is more effective for the achievement of outcomes than LBL. This is apparent in the students’ generally positive views towards the flipped classroom. However, interestingly, students also held positive views towards the LBL method with no significant difference between the levels of positivity. The positive points regarding the flipped classroom included general agreement that the flipped classroom stimulated interest in learning and guided self-study. Personalized learning, which is a prominent feature of the flipped classroom, also received recognition from most students.

Previous studies [7, 10–14] revealed that the flipped classroom significantly enabled students to acquire knowledge since the learning effects of the flipped classroom were more desirable than those of the LBL method. This study’s results agree with these findings. This study also confirmed that the flipped classroom gave students an advantage in knowledge acquisition. The following are possible reasons. First, this study revealed that the students in the flipped classroom group invested more time and effort than those in the LBL group. For example, the learning methods used in the flipped classroom group were more diverse. This increased investment in learning may have been an important factor for the higher level of understanding achieved by the experimental group [10, 14]. However, more than half of the flipped classroom group students agreed that they had to spend an increased amount of time and effort for learning via this method. Given the vastness of the medical curriculum, which may result in the increased time spent by students learning particular content to reduce the time spent on another part of the curriculum. This is probably the reason for the positive attitude that students have towards LBL since LBL requires less time. This raises questions about the role of the flipped classroom and could probably explain why students are glad to participate in flipped classroom learning.

The reasons for the satisfaction expressed by the students with the flipped classroom could be the following. First, the flipped classroom method of learning shifts the process of delivering knowledge prior to class, thereby providing the students an opportunity for personalized learning before and after class. The students can adjust their learning patterns according to their habits of perception and learning status. The classroom time aids integration of knowledge through student-teacher interactions and discussions between students. The flipped classroom increases the students’ interest in learning, improves their sense of self-control and guides self-study. These advantages are consistent with the findings of previous studies [11, 14, 17]. Exploring one’s potential for learning, meeting individual learning needs, and enhancing self-learning are possible reasons why students are generally satisfied with the flipped classroom. Secondly, in the LBL method, the instructor pays attention to the needs of the majority of the class and ignores individual differences between students. The flipped classroom enables personalized learning, and allows students with different learning habits and abilities to manage their learning rhythms and focus their learning according to their own specific situation. This boosts the cognitive characteristics of each student and contributes to students achieving better results [11]. Thirdly, the flipped classroom assigns relatively low-level cognitive learning, such as memorizing and understanding, outside of the classroom. In the classroom, teaching is accomplished mostly through teacher-student interactions and cooperation between peers, thereby stimulating the students’ intellectual potential [7, 12, 18]. The time in the classroom is used to achieve high-level cognitive learning skills, such as analytical thinking, critical thinking, and problem solving [7, 10, 17]. These factors are important reasons due to which students in the flipped classroom outperformed those in the control group.

Interestingly, the questionnaire revealed that the flipped classroom could be used as the most helpful learning tools. Their roles were significantly larger than their roles in the peer discussion. This could be because of the possibility that the students were generally more dependent on traditional lecture-style teaching and lacked confidence in their ability to implement self-learning and peer coaching. Simultaneously, the students also thought that the flipped classroom lacked systematic knowledge transfer methods compared to LBL. The students approved of the flipped classroom to the same extent as LBL. This result indicates that eliminating dependence on the teacher and changing learning modes are both challenges for Chinese students who are accustomed to passively receiving information. Respecting the authority of the lecturer leads to distrust in a new learning model that lacks systematic explanation and instead relies on teacher-student interactions. Although the students in the flipped classroom group achieved remarkably higher scores in the knowledge acquisition test, they nevertheless did not entirely approve of the new teaching model. This finding indicates that a significant amount of work needs to be done to change students’ ideas and habits about learning. Additionally, this study was administered at the end of the semester before the exam week, and the flipped classroom required a greater time investment and increased the study load. These could also be reasons why the flipped classroom did not receive a higher level of approval and recognition from the students. These results suggest that there are potential risks associated with the complete replacement of LBL with the flipped classroom in China. In many cases, the educator may need to apply different teaching methods customized to the curriculum and student characteristics.

Although interviews were conducted to investigate the teachers’ attitudes towards the flipped classroom, including the factors of teaching investment and evaluation, the reliability of the results needs to be considered due to differences in the subjective experiences of the teachers. Moreover, this was the first time that we had engaged in flipped classroom teaching; therefore, our teaching skills were not yet well developed, and the advantages of the flipped classroom were not fully reflected in what we offered. Overall, in this study, although teachers invested more time and energy into the flipped classroom, they also evaluated it to have greater learning effects. Future studies can attempt to study the impact of the flipped classroom on teachers.

## Conclusions

### Advantages of this study

The present study is the first to use the flipped classroom teaching approach for the ECG chapter of Medical Diagnosis in China. This study is innovative and possesses a certain reference value. Furthermore, in this study, the students performed better in the flipped classroom, and the students positively evaluated the flipped classroom model. This study can be used to further promote the research and application of new teaching models.

### Limitations of this study

First, due to the limited teaching hours and the proximity to the end of the term, this study tested a relatively small amount of course content. Therefore, the results should be generalized with caution. Further large-scale and more in-depth studies are needed to verify these results. Second, this study did not use heterogeneous grouping, and therefore, we could not validate similarities in academic levels and personality traits between students in each of the two groups. Therefore, balance within groups was not ensured, which might have resulted in in-group differences regarding experiences and achievements during the teaching process. This is a shortcoming of the study design. Third, we only set one time point (one week after class) to test the acquisition of knowledge, which is an insufficient amount of time to investigate the longevity of the retained knowledge. It would be worth repeating the test in the future to identify the potential long-term gains of this approach.

## Additional file

Additional file 1: Interview questionnaire on teacher’s attitudes towards the two pedagogics. It is semi-structured interview questionnaire of teachers to understand their experiences of both the flipped classroom and LBL method. Twenty teachers who participated in the interviews talked about the time they spent and other learnings. In addition, they were asked to provide a subjective evaluation of the teaching atmosphere and the perceived effects of the respective teaching method they used. For data collection and analysis, all these information were also recorded using videos. (DOCX 20 kb)

## Abbreviations

CAI: Computer-assisted instruction
ECG: Electrocardiogram
LBL: Lecture-based learning
RCT: Randomized controlled trial

## References

[1] Hattie J (2008). Visible learning: a synthesis of over 800 meta-analyses relating to achievement.

[2] Schwerdt G, Wupperman AC (2010). Is traditional teaching really all that bad? A within-student between-subject approach. Econ Educ Rev.

[3] Mattis KV (2015). Flipped classroom versus traditional textbook instruction: assessing accuracy and mental effort at different levels of mathematical complexity. Technol Knowl Learn.

[4] Farley J, Risko EF, Kingstone A (2013). Everyday attention and lecture retention: the effects of time, fidgeting, and mind wandering. Front Psychol.

[5] Lage M, Platt G, Treglia M (2000). Inverting the classroom: a gateway to creating an inclusive learning environment. J Econ Edu.

[6] Baker JW (2000). The Classroom flip: using web course management tools to become the guide by the side chambers.11th International Conference on College Teaching and Learning.

[7] van Vliet EA, Winnips JC, Brouwer N (2015). Flipped-class pedagogy enhances student metacognition and collaborative-learning strategies in higher education but effect does not persist. CBE Life Sci Educ.

[8] Bossaer JB, Panus P, Stewart DW, Hagemeier NE, George J (2016). Student performance in a pharmacotherapy oncology module before and after flipping the classroom. Am J Pharm Educ.

[9] Gorres-Martens BK, Segovia AR, Pfefer MT (2016). Positive outcomes increase over time with the implementation of a semiflipped teaching model. Adv Physiol Educ.

[10] Gross D, Pietri ES, Anderson G, Moyano-Camihort K, Graham MJ (2015). Increased preclass preparation underlies student outcome improvement in the flipped classroom. CBE Life Sci Educ.

[11] Veeramani R, Madhugiri VS, Chand P (2015). Perception of MBBS students to “flipped class room” approach in neuroanatomy module. Anat Cell Biol.

[12] Munson A, Pierce R (2015). Flipping content to improve student examination performance in a pharmacogenomics course. Am J Pharm Educ.

[13] Sharma N, Lau CS, Doherty I, Harbutt D (2015). How we flipped the medical classroom. Med Teach.

[14] Wakabayashi N (2015). Flipped classroom as a strategy to enhance active learning. Kokubyo Gakkai Zasshi.

[15] Zeng R (2015). Graphics-sequenced interpretation of ECG.

[16] Likert R (1932). A technique for the measurement of attitudes. Arch Psychol.

[17] McLaughlin JE, Griffin LM, Esserman DA, Davidson CA, Glatt DM, Roth MT, Gharkholonarehe N, Mumper RJ (2013). Pharmacy student engagement, performance, and perception in a flipped satellite classroom. Am J Pharm Educ.

[18] Moraros J, Islam A, Yu S, Banow R, Schindelka B (2015). Flipping for success: evaluating the effectiveness of a novel teaching approach in a graduate level setting. BMC Med Educ.

