# Supplementary material for: Friend or Foe? Flipped Classroom for Undergraduate Electrocardiogram Learning: a Randomized Controlled Study
Source: BMC Med Educ. 2017 Mar 7;17:53. doi: 10.1186/s12909-017-0881-8 (PMC5341445; doi:10.1186/s12909-017-0881-8)
Supplement: Additional file 1: — Interview questionnaire on teacher’s attitudes towards the two pedagogics. It is semi-structured interview questionnaire of teachers to understand their experiences of both the flipped classroom and LBL method. Twenty teachers who participated in the interviews talked about the time they spent and other learnings. In addition, they were asked to provide a subjective evaluation of the teaching atmosphere and the perceived effects of the respective teaching method they used. For data collection and analysis, all these information were also recorded using videos. (DOCX 20 kb) [file 12909_2017_881_MOESM1_ESM.docx]

**Interview questionnaire on teacher’s attitudes towards the two pedagogics**

**Hello, dear teachers! In order to understand your attitudes towards the flipped classroom and investment in the two pedagogics, would you like to spare a few minutes to answer the following questions in this questionnaire carefully and authentically! For data collection and analysis, your answers will be also kept in the video recordings of the interviews. We will strongly inform you that your answers will be kept strictly confidential! Thank you very much for your support and cooperation!**

1.Your personal information:

| Name: |  |
| --- | --- |
| Gender: |  |
| Title: |  |
| Age : |  |

**2**. How much time did you spent in the preparatory stage of the flipped classroom? (Please specific your answer, like hours on average you spent)

**3.** Have you ever visited many other medical universities in China or abroad in order to learn how to organize flipped classroom activities, or participated in several seminars about flipped classroom?

A、Yes, please clarify the specific forms, include the name of the universities, types of learning trainings, how you participated? and so on.

B、No.

**4.** How long is the average time **before** the class in the classroom stage of the flipped classroom teaching? (Please specific your answer, like hours on average you spent)

**5.** How long is the average time **after** the class in the classroom stage of the flipped classroom teaching? (Please specific your answer, like hours on average you spent)

**6.** How long is the average time in the preparatory and class stage of LBL? (Please specific your answer, like hours on average you spent)

**7.** Which teaching method cost you more time and energy? Please also give your reasons for this answer.

A、The flipped classroom

B、The LBL

**8.** What`s your opinions about the flipped classroom teaching? (Included the atmosphere, interest, and enthusiasm towards learning, and so on)

**9.** Do you think the flipped classroom is feasible and worth widely promoted in diagnostics teaching process? Please also give your reasons for this answer.

A、Yes, it is feasible and worth widely promoted.

B、No, it is not feasible and worth widely promoted.

C、I have no idea for this question.

**Thank you very much for your patience and sincere cooperation!**

**The flipped classroom investigation research group**
